# The impact of COVID-19 on the employment status and psychological expectations of college graduates: Empirical evidence from the survey data of Chinese recruitment websites

**DOI:** 10.3389/fpsyg.2022.1039945

**Published:** 2022-11-11

**Authors:** Yufei Mao, Yuan Zhang, Jiaxin Bai, Liangbo Zhang, Wenxin Hu

**Affiliations:** ^1^School of Labor Economics, Capital University of Economics and Business, Beijing, China; ^2^School of Economics and Management, Beijing Institute of Petrochemical Technology, Beijing, China; ^3^Human Resources Department, Beijing Machinery Industry Automation Research Institute Co., Ltd., Beijing, China

**Keywords:** COVID-19 epidemic, college graduates, employment status, psychological expectations, big data

## Abstract

Based on the big data and survey data of online recruitment platform, this paper empirically tests the impact of COVID-19 on the employment status and psychological expectations of college graduates. The results show that: under the impact of COVID-19 epidemic, both supply and demand sides of college graduates’ employment market are affected, such as the decline of recruitment demand, the rise of the employment supply, and the obvious decrease of employment market prosperity. The impacts of COVID-19 epidemic on college graduates’ employment status and psychological expectation in different cities are heterogeneous. In the short term, the epidemic has a negative impact on the employment of graduates, but the employment situation is gradually improving with the support of national policies. Under the influence of COVID-19 epidemic, graduates will change their employment location and expected salary, and they tend to choose “temporary non-employment,” and their proportions of getting offers and signing contracts are significantly reduced. This paper suggests: Firstly, we should continue to push forward the action plan of “expanding jobs in graduation season to promote employment,” and strengthen the persistence and permanence of employment promotion policies for college graduates; Secondly, encourage college students to change their employment concept and rationally adjust their employment expectations; Thirdly, to promote the development of flexible employment of college graduates, it is necessary to strengthen the propaganda of flexible employment, so that students can understand relevant policies; Fourthly, strengthen employment guidance services for graduates from poor families to ensure the continuity and stability of employment assistance policies.

## Introduction

College graduates are the groups with high human capital in the labor market, and their employment situations and psychological expectations have become the focus issues for the whole society (Li and Matlay, 2005; Ogunlel, 2012; Poon et al., 2021). The outbreak of COVID-19 epidemic in early 2020 not only has a great impact on China’s social economy, but also has a great impact on the employment status and psychological expectations of college graduates (Natali, 2022). Due to the expansion of enrollment in colleges and universities over the years, the number of college graduates in China exceeded 10 million for the first time in 2022, reaching 10.76 million. In addition, there was a sudden epidemic in some areas, and the employment of graduates faced multiple problems, such as supply pressure, tight demand, and structural contradiction. According to the *Report on the Employment Market Prosperity of College Graduates in the Second Quarter of 2022*, which was released by the China Institute for Employment Research of Renmin University of China, the COVID-19 epidemic broke out in some areas in April of 2022, especially in the Yangtze River Delta and Beijing–Tianjin–Hebei cities with large employment volume, which resulted in tight demand of enterprises, blocked recruitment of employers and prolonged job search period for job seekers. Theoretically speaking, the COVID-19 epidemic has a wide range of influences on college graduates, which not only reduces their employment opportunities and hinders their job search channels, but also leads to prolonged job search and increased psychological pressure (Esteban et al., 2020). Especially for graduates who cannot go back to school in time during the epidemic, and they can only search for jobs online, which undoubtedly leads to further increase of employment difficulty (Chen and Zhang, 2020; Li, 2020).

In 2022, China’s *Government Work Report* pointed out that there are more than 10 million college graduates this year, so it is necessary to strengthen employment and entrepreneurship guidance, policy support, and continuous service. In order to ensure the employment stability during the epidemic, the Ministry of Education, the Ministry of Human Resources and Social Security, the Ministry of Civil Affairs, and other departments jointly implemented policies to promote the employment of college graduates by promoting enterprises to stabilize their posts and expand employment, providing online employment services, and promoting entrepreneurship to drive employment. Thus, the impact of the COVID-19 epidemic on the employment of college graduates has become the attention focus for the society. At present, due to the data limitation, the research in this field is still mainly based on qualitative discussion or investigation and analysis (Cho et al., 2020; Jiang, 2021; Wang et al., 2021), especially the research on quantitative analysis by using network big data is scarce. What changes have taken place for the employment situation of college graduates under the impact of COVID-19 epidemic? What are the impacts of COVID-19 epidemic on graduates’ employment status and psychological expectations? What changes have been made to graduates’ job expectations and employment results? This paper will use the data of online recruitment platform to analyze the above problems.

The contributions of this paper are the following aspects: First, it enriches the relevant research on the relationship between COVID-19 epidemic, graduates’ employment, and psychological expectations. At present, many scholars at home and abroad have conducted rich discussions on COVID-19 epidemic and employment. Most literature focuses on overall employment situation, but less research concerns on specific group of college graduates. Different from the research perspective in previous studies, this paper focuses on the impact of epidemic on the employment in combination with the actual situation of college graduates, and puts forward hypotheses based on existing theories, which enriches the research in related fields. Second, different from the previous studies, which describes the impact of the epidemic on the employment of college graduates from a qualitative perspective, this paper empirically tests the direction, degree, and scope of the impact of COVID-19 epidemic on graduates’ employment by quantitative method, and discusses its heterogeneity and time trend effect. Third, due to the limitation of enterprise data, the existing literature mostly focuses on the supply side of college graduates to analyze the impact of their job-hunting experience and employment destination. This paper not only discusses the changes of graduates’ employment supply and demand under the impact of epidemic situation, but also analyzes the impact of epidemic situation on the matching results of job market. Fourth, in terms of research data, most traditional studies use administrative record data or survey data to analyze the employment problem of college graduates. These data have some shortcomings, such as strong subjectivity, limited survey frequency, and non-sampling error. The network big data used in this paper is massive, timely, objective, and authentic, which can make up for the shortcomings of traditional research.

## Analysis of employment situation

In order to objectively describe the employment situation of college graduates under the impact of COVID-19 epidemic situation, this paper uses the big data of Zhaopin recruitment network to analyze recruitment demand, graduates’ job supply, and employment market prosperity. It should be noted that the data used in this paper is not crawled from the Internet, but directly extracted from the background of Zhaopin recruitment website. According to the existing policies and regulations, unemployed college graduates who leave school within 2 years can keep their household registration in the school, or transfer them to the public employment talent service institutions, and they are allowed to take part in the examination for civil servants and public institutions as fresh graduates. Therefore, all job seekers with college degree or above and graduation time within 2 years (with June as the boundary) are regarded as the supply side of college graduates, the positions with “fresh graduates” in the recruitment requirements are regarded as the demand side of college graduates. Then, the data of supply and demand are correspondingly matched.

**Figure 1:**
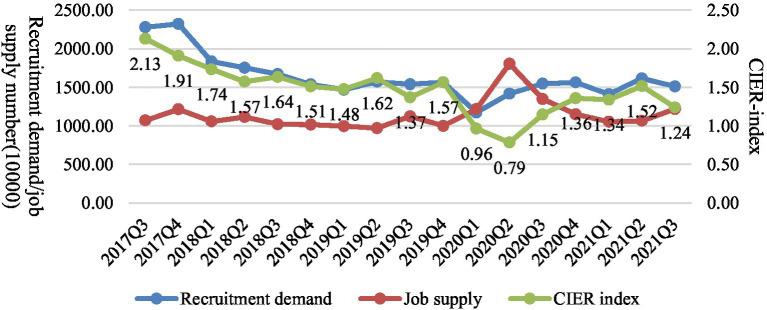
Employment trend of college graduates from 2018 to 2021.

Figure 1 reports the employment supply and demand of graduates monthly, and points out the changing trend of employment prosperity index. Among them, the employment prosperity index refers to the CIER index construction method of China Institute for Employment Research of Renmin University of China (Zeng, 2015), and it is calculated by using the ratio of the number of recruitment demand to the number of job supply. When the value is greater than 1, it indicates that the job market demand exceeds supply, and the employment prosperity degree is high. On the contrary, if the value is less than 1, it indicates that the job supply exceeds job demand, and the degree of employment prosperity is low. Previous studies have confirmed that CIER index is closely related to macroeconomic fluctuations, and it can be used as an important indicator to reflect changes in the job market (Geng and Mao, 2017). This paper uses this index to analyze the employment market of college graduates, which is scientific and expands the research in this field. As for the demand side, under the impact of COVID-19 epidemic, the recruitment demand of enterprises in 2020 showed a trend of first decreasing and then rising, but after the second quarter of 2021, the number of recruitment demand continued to decline. As for the supply side, in the first quarter of 2020, the number of online job-seeking increased obviously, and then gradually fell back and stabilized. However, after entering 2021, the number of job-seeking supply continued to rise, which increased the pressure on the supply side. From the perspective of the employment prosperity index, there was a continuous decline in the first and second quarters of 2020. However, with the improvement of domestic epidemic prevention and the strong support of national policies, the prosperity index rose after the third quarter. After the second quarter of 2021, due to the double effects of increased supply and tight demand, the prosperity index gradually declined.

**Figure 2:**
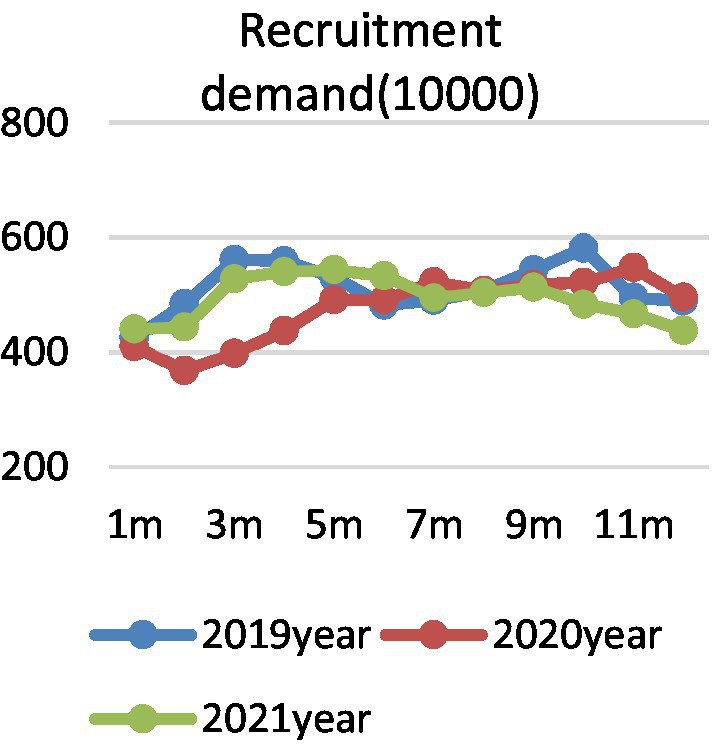
Changes in recruitment demand.

**Figure 3:**
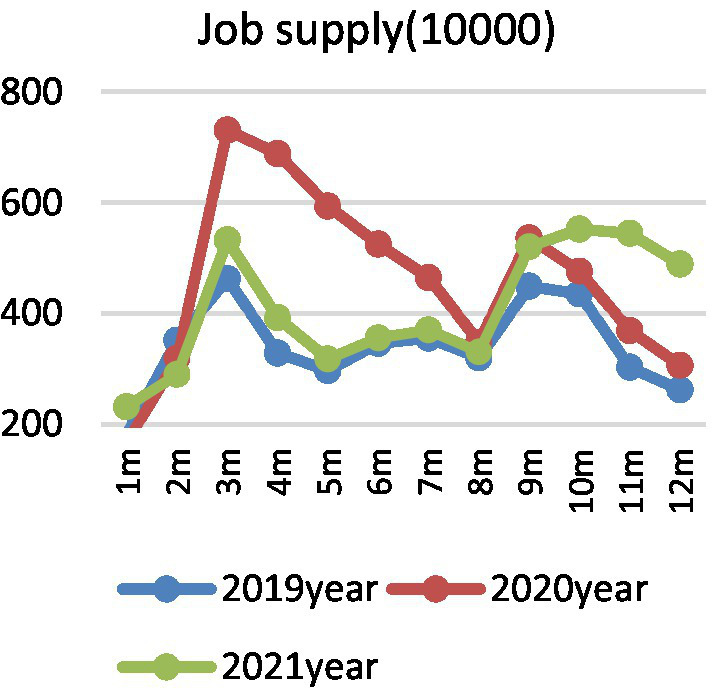
Changes in job supply.

**Figure 4:**
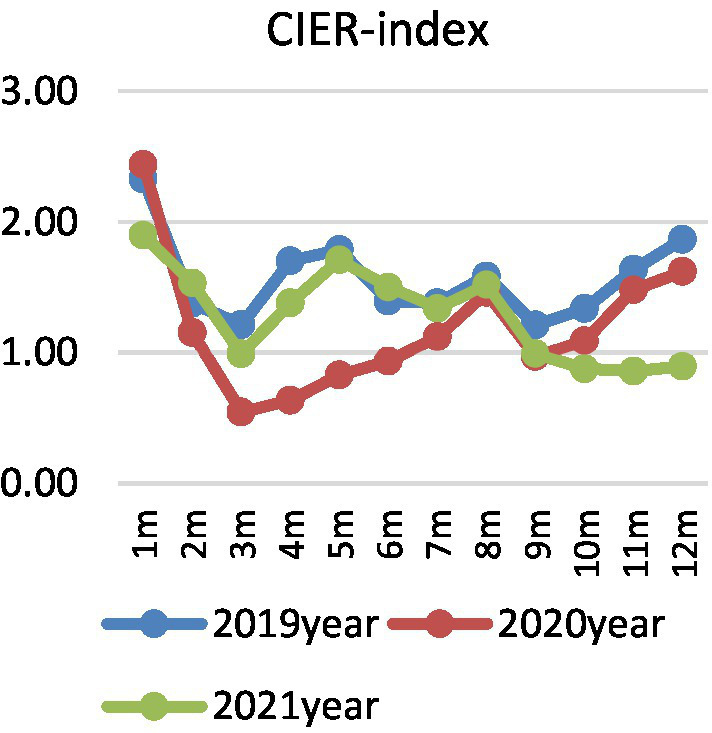
Changes in employment prosperity index.

In view of the seasonal characteristics of college graduates’ employment market, quantitative description cannot eliminate the interference of seasonal factors. Therefore, this paper also draws the year-on-year changes of supply, demand, and employment prosperity index of college graduates in different months of 2019, 2020, and 2021. Figure 2 shows the demand side of college graduates, there is a “U”-shaped trend from January to August in 2020, and it declines and then increases. Affected by the epidemic situation, the recruitment demand of enterprises for college graduates is obviously lower than that of the same period in 2019, reaching the lowest level in March, then gradually rising, and steadily declining after entering July. In fact, different from the short-term, structural and local effects of the SARS epidemic in 2003, COVID-19 epidemic affected a wide range, which not only caused a significant impact on the social economy, but also caused difficulties in rework, shutdown, and production stoppage of enterprises due to its overlap with the Spring Festival holiday, which seriously affected the employment demand (Gao, 2020; Mo et al., 2020). Especially for tourism, aviation, hotel industry, and some small and medium-sized enterprises with poor anti-risk ability, the recruitment demand dropped sharply, and even there was a crisis of layoffs (George and Hugh, 2020). However, after the epidemic prevention, the overall trend returned to before the epidemic, and then gradually declined after September. Figure 3 shows the supply side of college graduates. The number of online job seekers of college graduates represents an inverted “U” trend over time. From March to May in 2020, the number of job seekers of graduates increased by more than 150% year-on-year, and gradually stabilized after July. Considering the reality, the number of college graduates in China in 2020 is 8.74 million, and in 2021 is 9.09 million, respectively, together with some returned overseas students, which undoubtedly causes a large pressure of concentrated employment. Affected by the COVID-19 epidemic, on the one hand, graduates’ previous job-seeking period in spring has also been postponed accordingly. On the other hand, due to the requirements of epidemic prevention and control, offline campus recruitment turned into online job hunting, resulting in a significant increase in the number of online job seekers year-on-year. Figure 4 shows the employment prosperity index of college graduates. The employment prosperity index from January to September 2020 showed a trend of first decreasing and then increasing, and it reached the lowest level in April, that is, the competition was the fiercest. After May, with the support of China’s epidemic prevention and national policies, the employment prosperity also gradually picked up. In 2021, the employment prosperity index returned to before the epidemic, but after August, the employment prosperity index was lower than the same period.

## Empirical analysis

Studies have shown that unexpected events of epidemic will not only affect economic growth, but also have an impact on employment scale, unemployment rate, and job market (Laura et al., 2020; Murillo et al., 2020; Bai et al., 2022). In order to test the impact of epidemic impact on college graduates’ employment, this paper, based on the matching of online recruitment big data and statistical yearbook data, empirically tests the direction and degree of epidemic impact on graduates’ employment, and discusses its heterogeneity and time trend effect.

### Variable selection and description statistics

The data used in this paper comes from 2019 to 2020 network big data of 55 core cities recruited by Zhaopin Limited Company. The variables explained in this paper are the recruitment demand, job supply, and employment prosperity of college graduates at the urban level. The college graduates cover different educational backgrounds, school levels, and majors. In addition, variables such as city level, administrative region, urban population size, *per capita* GDP, fiscal expenditure, number of employees and students on the job, industrial structure, and proportion of domestic-funded enterprises are also added. This part of data is mainly compiled according to the 2020 China Urban Statistical Yearbook and other data. Description and description statistics of variables are shown in Table 1. According to the monthly data from January to September 2019 and 2020, a total of 990 samples were obtained. Regarding the core explanatory variable epidemic impact, it is measured before and after the outbreak, in which the value before the outbreak (2019) is 0, and the value after the outbreak (2020) is 1. As for the city levels, the samples include four first-tier cities in Beijing, Shanghai, Guangzhou, and Shenzhen; 15 new first-tier cities in Chengdu, Dalian, Dongguan, Hangzhou, Nanjing, Ningbo, Qingdao, Shenyang, Suzhou, Tianjin, Wuhan, Xi’an, Changsha, Zhengzhou, and Chongqing; 25 s-tier cities in Changzhou, Foshan, Fuzhou, Guiyang, Harbin, Hefei, Huizhou, Jinan, Jiaxing, Kunming, Nanchang, Nanning, Nantong, Quanzhou, Shijiazhuang, Taiyuan, Weifang, Wenzhou, Wuxi, Xuzhou, Yantai, Yangzhou, Changchun, Zhongshan, and Zhuhai; 11 third-tier cities including Baotou, Daqing, Hohhot, Huai’an, Linyi, Luoyang, Qinhuangdao, Weihai, Xianyang, Yangzhou, and Zhenjiang.

**Table 1:** Variables description and description statistics.

Variable name		Variable definition	Observations	Mean	Std.
Recruitment demand	Demand position	Number of positions required for recruitment (10,000)	990	0.859	1.070
	Demand personnel	Number of personnel required for recruitment (10,000)	990	7.147	8.481
Job search supply	Number of suppliers	Number of online job seekers (10,000)	990	8.879	15.55
	Number of delivered	Number of online resumes delivered (10,000)	990	34.23	68.19
Employment boom		The ratio of the number of recruits to the number of job seekers	990	1.631	1.534
Epidemic impact		Before and after the outbreak of COVID-19 (2020 = 1, 2019 = 0)	990	0.500	0.500
Urban characteristics	City grade	First-tier cities (yes = 1, no = 0)	990	0.073	0.260
		New first-tier cities (yes = 1, no = 0)	990	0.273	0.446
		Second-tier cities (yes = 1, no = 0)	990	0.455	0.498
		Third-line city (yes = 1, no = 0)	990	0.200	0.400
	Administrative area	Eastern region (yes = 1, no = 0)	990	0.618	0.486
		Central region (yes = 1, no = 0)	990	0.127	0.333
		Western region (yes = 1, no = 0)	990	0.164	0.370
		Northeast region (yes = 1, no = 0)	990	0.091	0.288
	Population size	Average population at the end of the year (10,000 people)	990	700.1	483.7
	*Per capita* GDP	*Per capita* GDP (10,000 yuan)	990	10.58	3.378
	Fiscal expenditure	Local public budget expenditure (100 million yuan)	990	1335	1539
	On-duty worker	Average number of employees on the job (10,000)	990	152.2	135.8
	Number of students	Number of general college students (10,000)	990	33.79	27.44
	Industrial structure	The proportion of secondary and tertiary industries in GDP	990	0.959	0.030
	Enterprise operated on domestic capital	The proportion of domestic-funded enterprises in industrial enterprises	990	0.834	0.116

According to the big data of Zhaopin.com and the data of China Urban Statistical Yearbook 2020. Cities are classified according to the list published by China Business News.

### Measurement model and empirical results

In order to explore the net effect of COVID-19 epidemic on graduates’ employment, on the basis of previous research literature on graduates’ employment (Li and Matlay, 2005; Ogunlel, 2012), this paper expands existing research practices to build the related model. The software used in the analysis is Stata 14. Considering that the employment of college graduates is affected by many factors, in order to eliminate the interference of these factors, this paper uses a seemingly unrelated regression model to estimate the impact of epidemic, as shown in formula (1):


(1) $$\{\begin{array}{c} Demand=\alpha_{0}+\alpha_{1}COVID19+\beta_{i}city_{i}+\gamma_{i}month_{i}+\varepsilon \\ Supply=\alpha_{0}+\alpha_{1}COVID19+\beta_{i}city_{i}+\gamma_{i}month_{i}+\varepsilon \\ CIER=\alpha_{0}+\alpha_{1}COVID19+\beta_{i}city_{i}+\gamma_{i}month_{i}+\varepsilon \end{array}$$

In formula (1), Demand, Supply, and CIER are, respectively, the employment recruitment demand (the number of required positions and the number of required positions), job search supply (the number of supply and delivery times), and employment prosperity of college graduates; COVID-19 is the epidemic impact variable, and *α_i_* is the impact coefficient of epidemic impact. *city_i_* is the control variable at the city level; *month_i_* is the monthly time variable; *ε* is a random disturbance term.

After the outbreak of the epidemic, the Chinese government has issued a series of policies to promote the employment of college graduates, and the implementation effect of these policies will gradually appear with the passage of time, which will bring about new changes in the job market. To investigate this time trend effect, this paper introduces the interaction term of epidemic impact (COVID-19) and monthly time variable (*month_i_*) into the benchmark model, as shown in formula (2):


(2) $$\begin{array}{l} E_{j}=\alpha_{0}+\alpha_{1}COVID-19+\alpha_{i}COVID-19\times month_{i}\\ +\beta_{i}X_{i}+\varepsilon \end{array}$$

Columns (1) and (2) of Table 2 show the estimated results of the number of jobs and the number of people in need affected by the epidemic impact, respectively. After adding the control variables, the coefficients of epidemic impact are −0.213 and −0.848, respectively, and both of them are significant at the level of 1%, which indicates that after excluding other factors, due to the epidemic impact, the number of jobs and the number of recruitment needs of enterprises in various cities have decreased significantly. This is because, from the demand point of view, due to the epidemic prevention and control requirements of the Chinese government, most enterprises in cities have to stop production and work, especially in the personnel-intensive industries, such as tourism, aviation, hotels, etc., the demand for recruitment has dropped significantly. Columns (3) and (4) are the supply number and delivery times, and their coefficients are 1.59 and 2.14, respectively, and both of them are significant at the level of 1%, that is, the supply number of college graduates’ job hunting has increased significantly. This is because the job-hunting process of college graduates has changed from offline to online, which leads to an increase in the number of online job applicants. In addition, the cost of online job hunting is low. In order to improve the interview opportunities when the graduation season approaches, job seekers choose to deliver jobs to multiple cities at the same time. Column (5) shows the impacts of epidemic on employment prosperity. The results showed that the epidemic coefficient was −0.256 and was significant at the level of 1%, which indicated that after the epidemic, the employment prosperity of college graduates declined obviously due to the double influence of the decline of recruitment demand and the increase of job-seeking supply. In terms of control variables, in terms of city level, with first-tier cities as the reference group, the recruitment demand and job supply of new first-tier, second-tier and third-tier enterprises are obviously less, but the employment boom of new first-tier cities is slightly higher than that of first-tier cities. Administratively, taking the eastern region as the reference group, the recruitment demand and employment boom in the central region are obviously higher, while the number of job applicants is relatively small; the number of job applicants in the western region is relatively small; and the recruitment demand and employment boom in Northeast China are relatively low.

**Table 2:** Impact of epidemic on employment of college graduates.

Variable		Recruitment needs		Job search supply		Employment boom
		(1)	(2)	(3)	(4)	(5)
		Number of required positions	Demand number	Number of suppliers	Delivery times	CIER-index
Epidemic impact		−0.213^***^ (0.025)	−0.848^***^ (0.215)	1.59^***^ (0.519)	2.14^***^ (0.439)	−0.256^***^ (0.066)
City grade	New first-tier city	−1.772^***^ (0.108)	−12.104^***^ (0.858)	−27.587^***^ (2.722)	−121.811^***^ (12.748)	0.234 (0.148)
	Second-tier city	−2.089^***^ (0.127)	−14.491^***^ (1.016)	−28.944^***^ (2.875)	−123.634^***^ (13.152)	−0.059 (0.189)
	Third-tier city	−1.874^***^ (0.152)	−12.268^***^ (1.231)	−23.965^***^ (3.176)	−100.168^***^ (14.289)	−0.071 (0.252)
Administrative area	Central region	0.154^***^ (0.05)	2.598^***^ (0.471)	−3.886^***^ (0.878)	−16.758^***^ (4.001)	0.925^***^ (0.144)
	Western region	−0.075 (0.051)	0.211 (0.48)	−0.833 (0.968)	−4.667 (4.24)	0.056 (0.152)
	North-east region	−0.072^***^ (0.027)	−0.436^**^ (0.193)	−0.189 (0.546)	−0.017 (2.371)	−0.169 (0.125)
Urban characteristics		Control	Control	Control	Control	Control
Time trend		Control	Control	Control	Control	Control
Constant term		−1.346^**^ (0.678)	−23.656^***^ (5.039)	−59.844^***^ (12.033)	−237.319^***^ (53.796)	15.322^***^ (3.126)
Variance ratio		212.79	204.57	49.73	42.11	39.61
R^2^		0.869	0.845	0.730	0.691	0.548
Observations		990	990	990	990	990

According to the big data of Zhilian Recruitment Network and the data authors of China Urban Statistical Yearbook 2019. Marginal effects are reported in the table, and robust standard errors are shown in parentheses.

*** Significant at the level of 1%.

** Significant at the level of 5%.

* Significant at the level of 10%.

In view of the seasonal characteristics of the employment market of college graduates, and after the outbreak of the epidemic, the state issued a series of policies to promote the employment of graduates, which can cushion the negative impact of the epidemic to a certain extent. In addition, in the long run, during the epidemic, people formed online office habits, which will lead to new employment opportunities, such as live webcast and online education, which is conducive to college students’ diversified employment choices. In order to identify this time trend effect, this paper takes September as the reference group, and introduces the interaction between epidemic impact and monthly time variables, and the empirical results are shown in Table 3. From the perspective of recruitment demand, the interaction coefficient between epidemic impact and February–April is significantly negative, while the interaction coefficient with other months is not significant. It shows that after the outbreak of the epidemic, the recruitment demand of college graduates decreased obviously from February to April, and then gradually stabilized. Columns (3) and (4) from the perspective of job supply, the interaction coefficient between epidemic impact and February and July is significantly negative, and that between March and May is significantly positive. This shows that the impact of the epidemic first brought a significant negative impact on college graduate supply. Then, with the promotion of online job hunting and the arrival of college graduation season, more and more graduates posted their resumes through the Internet, which led to a significant increase in the number of job applicants, which did not begin to decline until July. Column (5) the interaction coefficient between the epidemic impact and January–February and June–August was significantly positive, while the interaction coefficient with March–May was significantly negative. According to the reality, from January to February, the epidemic impact has a negative impact on both the supply and demand sides of college graduates’ employment, but the supply side has a larger decline, which makes the employment boom rise in a short time. From March to May, the impact of the epidemic caused the double factors of the decrease of enterprise recruitment demand and the increase of online job supply, resulting in a significant decline in the employment boom. From June to July, with the strong support of the national employment promotion policy and the continuous improvement of epidemic prevention and control, the employment prosperity of college graduates is also rising month by month.

**Table 3:** Time trend effect of epidemic.

Variable	Recruitment needs		Job search supply		Employment boom
	(1)	(2)	(3)	(4)	(5)
	Number of required positions	Demand number	prosperity index	Delivery times	Employment boom
Epidemic impact	−0.058 (0.071)	−0.132 (0.642)	1.061 (1.102)	−0.884 (5.485)	−0.488^***^ (0.109)
The epidemic × January	−0.097 (0.094)	−0.152 (0.823)	−2.097 (2.171)	−2.538 (9.963)	1.822^***^ (0.479)
The epidemic× February	−0.282^***^ (0.102)	−1.472^*^ (0.873)	−3.348^*^ (1.705)	−9.971 (8.151)	0.723^***^ (0.183)
The epidemic × March	−0.321^***^ (0.109)	−2.104^**^ (0.931)	5.374^**^ (2.31)	16.781 (10.42)	−0.472^***^ (0.146)
The epidemic × April	−0.235^**^ (0.106)	−1.598^*^ (0.96)	5.654^***^ (1.917)	21.408^**^ (8.838)	−0.796^***^ (0.166)
The epidemic × May	−0.183^*^ (0.104)	−0.751 (0.952)	3.631^**^ (1.72)	16.659^**^ (8.067)	−0.367^**^ (0.152)
The epidemic × June	−0.133 (0.102)	−0.012 (0.889)	0.965 (1.731)	5.842 (8.621)	0.253^*^ (0.147)
The epidemic × July	−0.088 (0.103)	−0.089 (0.919)	−3.462^*^ (2.06)	−13.687 (10.975)	0.548^***^ (0.138)
The epidemic × August	−0.05 (0.104)	−0.273 (0.915)	−1.957 (1.641)	−7.271 (8.469)	0.373^**^ (0.146)
Observations	990	990	990	990	990

According to the big data of Zhilian Recruitment Network and the data authors of China Urban Statistical Yearbook 2019. The regression coefficient of each variable is in the table, and the steady standard error is in brackets.

*** Mean significant at 1%.

** Mean significant at 5%.

* Mean significant at 10%.

## The micro mechanism analysis

The foregoing analysis shows that the impacts of COVID-19 are heterogenous. In the short term, the recruitment demand of enterprises decreases, while the number of online job applicants increases, and the employment boom of college graduates decreases obviously. However, under the buffer and intervention of relevant policies, the employment boom of college graduates is also gradually rising. So, what is the change of college graduates’ psychological expectation of job hunting under the influence of epidemic situation? This paper further analyzes this from a microscopic perspective.

### Variable selection and description statistics

This data comes from the online survey data of online recruitment platform. The survey time is from February to April 2020, and a total of 7,571 college graduates are collected. The purpose of this survey is to understand the changes of college graduates’ psychological expectations and employment choices after the outbreak of COVID-19. The questionnaire not only contains the related topics affected by the epidemic, but also includes the graduates’ personal characteristics, campus experience, psychological expectations, employment destination, and other issues. It should be noted that the sample of college graduates in this survey involves different educational groups, such as junior college, undergraduate, and graduate students, and covers 31 provinces and cities in China, which is representative to some extent.

The explained variables psychological expectations are mainly measured from employment location, expected salary, and expected industry, which, respectively, correspond to three topics in the questionnaire: change of employment location (yes = 1), expected salary decrease (yes = 1), and expected industry change (yes = 1). Regarding the perception of epidemic situation as the core explanatory variable, it is mainly measured by whether college graduates’ self-perception of job hunting is affected by the epidemic situation, corresponding to the questionnaire “Does the new pneumonia epidemic during the Spring Festival affect your job hunting?,” if “Yes” is selected, the value is 1; if “No,” the value is 0. In addition, existing studies have discussed the factors influencing graduates’ employment, including human capital, social capital, school characteristics, and family economic conditions (Lai et al., 2012). In order to eliminate the interference of these factors, this paper also controls the variables of college graduates’ campus experience, individual characteristics, educational level, major type, and family characteristics in the analysis. From the perspective of personal characteristics, in terms of gender ratio, the proportion of men is 41.9% and that of women is 58.1%; In terms of household registration type, rural household registration is 60.6%; Urban household registration is 39.4%. In terms of the distribution of college students’ academic qualifications, there are more undergraduate degrees, accounting for 74.4%, while junior college students and graduate students account for a relatively small proportion, accounting for 16.8 and 8.8%, respectively. From the distribution of majors, there are many majors in engineering, economics, and management, accounting for 33.1%, 28.9%, and 10.5% respectively, while there are relatively few majors in language, humanities, medicine, and law, accounting for 4.1%, 3.7%, 2.5%, and 1.6%, respectively. From the perspective of school types, the proportion of graduates from general undergraduate courses is the highest, accounting for 63.1%, while the proportion of graduates from junior colleges, double-top universities, and research institutes is 18.0%, 16.8%, and 2.2%, respectively. After deleting the samples with missing key variables, a total of 5,179 valid samples were retained. Among them, 62.9% thought that job hunting was affected by the epidemic, and 37.1% thought that job hunting was unaffected.

### Measurement model and empirical results

In this paper, probit model is used to analyze the impact of epidemic perception on college graduates’ employment psychological expectations. The first column in Table 4 is the estimated result. In this paper, the change of employment place, the decline of expected salary, and changes for expected industry are taken as the explained variables, and the coefficients of epidemic perception are 0.050, 0.174, and 0.084, respectively, all of these variables are significant at the level of 1%. Therefore, the graduates’ self-perception of being affected by the epidemic situation will also change their job-seeking expectations, which are mainly manifested in changing the place of employment, lowering the expected salary, and changing the expected industries. Considering that the influence of college graduates’ perception of epidemic situation on employment may have endogenous problems caused by self-selection bias, this paper uses the Propensity Score Matching (PSM) to test the impacts. Columns (2) to (4) in Table 4 show the estimation results obtained by using three methods: kernel matching, radius matching, and K-nearest neighbor matching in caliper. Taking the nuclear matching as an example, the average processing effect (ATT) coefficients of epidemic situation perception on employment place, expected salary, and expected industry are 0.049, 0.016, and 0.078, respectively, all of which are significant at the level of 1%. It can be seen that the coefficients obtained by using PSM are consistent with the previous ones, and the conclusion is robust.

**Table 4:** Influence coefficient of epidemic perception on employment of college graduates.

Variable	Probit model	Nuclear matching	Radius matching	K-nearest neighbor matching
**Panel A: the impact of epidemic perception on the expected employment place**				
Epidemic perception	0.050^***^ (0.013)	0.049^***^ (0.013)	0.049^***^ (0.013)	0.054^***^ (0.014)
Observations	5179	5179	5179	5179
**Panel B: the impact of epidemic perception on the decline of expected salary**				
Epidemic perception	0.174^***^ (0.013)	0.169^***^ (0.012)	0.170^***^ (0.012)	0.170^***^ (0.013)
Observations	5179	5179	5179	5179
**Panel C: the impact of epidemic perception on changes for expected industry**				
Epidemic perception	0.084^***^ (0.013)	0.078^***^ (0.013)	0.080^***^ (0.013)	0.079^***^ (0.014)
Observations	5179	5179	5179	5179

According to the online survey data of Zhaopin recruitment network platform. Marginal effects are reported in the table, and robust standard errors are shown in parentheses. In the regression, variables such as educational level, major type, family characteristics, and province are controlled. In the table above, the bandwidth of nuclear matching method is set to 0.01, the radius in radius matching is set to 0.005, and the number of K-nearest neighbor matching elements in caliper is 4.

*** Significant at the level of 1%.

** Significant at the level of 5%.

* Significant at the level of 10%.

## Discussion

Under the impact of the COVID-19, how to study and judge the changes in the employment market and psychological expectations of graduates in a timely manner and implement accurate employment policies in key regions and cities has become a topic of great concern to the government and all walks of life. This paper uses the data of the online recruitment platform to empirically test the impact effect and mechanism of the epidemic on the employment of college graduates from the macro and micro levels by constructing an econometric model. The statistical techniques used in the paper are ordinary least squares regression (OLS), probit model, and descriptive statistics and the software used in the empirical analysis is Stata 14. In summary, this research is an observational and empirical study, and it reviews the previous literature and puts forward new research conclusions.

The empirical results verify the following conclusions: first, the COVID-19 epidemic has impacted the employment market of college graduates, which is manifested in the obvious decline in the number of job vacancies and demands of enterprises, the obvious increase in the number and number of online job postings, and the significant decrease in the employment boom. Second, from the perspective of time trends, the impact of the epidemic will have a negative effect on the graduate employment market in the short term, but with policy intervention and comprehensive prevention and control, the graduate employment situation is also gradually warming up. Third, due to the impact of the epidemic, graduates’ employment psychological expectations will change significantly, mainly manifested in changing employment locations, adjusting expected salaries, and expected industries. The above conclusions mean that although the epidemic has a great impact on the employment of college graduates in the short term, the employment situation of graduates has gradually stabilized under the guidance of national policies. In the future, it is still necessary to pay more attention to the employment structure contradictions between different regions and cities and guide graduates to choose jobs rationally.

## Implications

Our analysis has some implications for employment and psychology of college graduates. Based on the research conclusions, this paper suggests: first, we should continue to promote the action plan of “expanding jobs and promoting employment in the graduation season.” In order to cope with the impact of the epidemic and other uncertainties, we suggest that we should strengthen the sustainability and permanence of the employment promotion policies for college graduates. Colleges and universities can make full use of Internet technology, improve the employment information release channels, such as the employment information network, WeChat official account, and employment QQ group, and promote enterprises to actively connect with colleges and universities, fully gather high-quality enterprise resources, and invite enterprises to enter the campus through the combination of online and offline. Second, college students should be encouraged to change their employment concepts and reasonably adjust their employment expectations. In the face of the overlapping environment of supply and demand differentiation in the college students’ employment market, employment guidance should be strengthened, and college students should be encouraged not only to focus on the immediate gains and losses, but also to change their employment concepts in time according to the employment situation and enterprise needs, reasonably adjust their employment expectations, and find a suitable career development path.

In order to promote the development of flexible employment of college graduates, we should strengthen the publicity of flexible employment, let students understand relevant policies, help them make rational choices in combination with their own characteristics, and evaluate the benefits and risks of flexible employment. The education department should closely follow the development trend of emerging industries, including flexible employment in majors, training programs, and curriculum settings, and closely link talent training with the needs of the labor market. The human resources and social security department should speed up the establishment of a healthy and flexible employment social security system, and provide social security subsidies, pension, and medical insurance for freelance college students to solve their worries. In addition, we should strengthen employment guidance services for graduates from difficult families and ensure the continuity and stability of employment assistance policies. Due to the narrow employment scope and single information channel of graduates with difficulties, it is recommended that universities and local human resources and social security departments continue to strengthen the provision of free policy consultation, employment guidance, career introduction, and other employment services for graduates with difficulties before and after they leave school, and make accurate and targeted recommendations in combination with the graduates’ job-hunting wishes.

## Data availability statement

The original contributions presented in the study are included in the article/supplementary material, further inquiries can be directed to the corresponding author.

## Ethics statement

Ethical review and approval were not required for the study on human participants in accordance with the local legislation and institutional requirements. The patients/participants provided their written informed consent to participate in this study.

## Author contributions

YM designed the theoretical model and wrote the original manuscript. YZ analyzed the data and improved the manuscript. JB is mainly responsible for the content modification of the paper. LZ is mainly responsible for the final draft revision. WH contributed to writing review and editing and revised and supervised the entire work. All authors contributed to the article and approved the submitted version.

## Funding

This research was supported by the Youth Program of Beijing Social Science Foundation (21JJC022), Research and Training Program for College Students (URT) of Beijing Institute of Petrochemical Technology (2021J00103), Cross-Disciplinary Science Foundation from Beijing Institute of Petrochemical Technology (project no. BIPTCSF-024), 2022–2023 Open Project of Development Research Centre of Beijing New Modern Industrial Area (JD2022001), and Scientific Research Program of Beijing Municipal Education Commission (SM202010038011).

## Conflict of interest

LZ was employed by company Beijing Machinery Industry Automation Research Institute Co., Ltd.

The remaining authors declare that the research was conducted in the absence of any commercial or financial relationships that could be construed as a potential conflict of interest.

## Publisher’s note

All claims expressed in this article are solely those of the authors and do not necessarily represent those of their affiliated organizations, or those of the publisher, the editors and the reviewers. Any product that may be evaluated in this article, or claim that may be made by its manufacturer, is not guaranteed or endorsed by the publisher.
